# Control Effects of *Chelonus munakatae* Against *Chilo suppressalis* and Impact on Greenhouse Gas Emissions From Paddy Fields

**DOI:** 10.3389/fpls.2020.00228

**Published:** 2020-03-06

**Authors:** Daijia Fan, Haoran Zhang, Tianqi Liu, Cougui Cao, Chengfang Li

**Affiliations:** ^1^MOA Key Laboratory of Crop Ecophysiology and Farming System in the Middle Reaches of the Yangtze River, College of Plant Science and Technology, Huazhong Agricultural University, Wuhan, China; ^2^Hubei Collaborative Innovation Center for Grain Industry, Yangtze University, Jingzhou, China

**Keywords:** *Chilo suppressalis*, *Chelonus munakatae*, CH_4_, N_2_O, paddy field

## Abstract

Field and pot experiments were conducted to investigate the control effects of parasitoid wasps (*Chelonus munakatae* Munakata) on striped rice stem borers and their impacts on N_2_O and CH_4_ emissions from paddy fields. Three treatments including no insect (NI), striped stem borer (CS) and parasitoid wasp + striped stem borer (CS+CM) were implemented. The abundance of GHG-related microorganisms in soils was determined by absolute real-time qPCR. Compared with NI, CS and CS+CM significantly increased the ratio of dead tillers, inhibited the growth and vitality of rice roots, and decreased the rice grain yield, while they significantly reduced the seasonal cumulative emissions of N_2_O and CH_4_ by 17.7–24.6 and 13.6–35.1%, and decreased the total seasonal global warming potential (GWP) by 13.6–34.7%, respectively. Moreover, compared with CS, CS+CM significantly enhanced the growth and vitality of rice roots, decreased the ratio of dead tillers, improved the rice grain yield, as well as increased the seasonal cumulative CH_4_ emissions and the total seasonal GWP. Principal component analysis indicated that the morphological features of rice roots play a more important role in regulating GHG emissions than GHG-related microorganisms. The results suggested that *C. munakatae* can effectively control the outbreak of *C. suppressalis* and alleviate crop damage with acceptably higher GHG emissions. It is concluded that it can be recommended as an effective, environment-friendly and sustainable approach to prevent and control *C. suppressalis*.

## Introduction

Striped rice stem borer (*Chilo suppressalis* Walker) is one of the major pests that severely affect rice production (Herdt, 1991). Currently, the control and prevention of stem borers mainly depend on chemical and biological approaches (Duan et al., 2012). Although chemical pesticides are highly effective, their long-term and extensive application involve a series of drawbacks such as high costs, pesticide resistance and environmental pollution (Duan et al., 2012). Moreover, the population of natural enemies of stem borers has drastically decreased due to environmental deterioration and cannot be restored naturally, which further aggravates the outbreak of stem borers (Duan et al., 2012). It has been generally recognized that biological diversity plays an important role in the sustainable development of agriculture (Kennedy and Smith, 1995; Brussaard et al., 2007). Among the various natural enemies of striped rice stem borers in China, parasitoid wasp (*Trioxys complanatus*) is the dominant species, which has been thoroughly studied and widely used as a secure and reliable method to prevent and control stem borers (Chen et al., 2010; Wang and Ma, 2015). In the 1970s, two species of *Trichogramma* wasps, *Trichogramma japonicum* (Ashmead) and *Trichogramma chilonis* (Ishii), were utilized for the control of striped rice stem borers in China, which had brought fairly satisfactory results in some areas (Huang et al., 2018). Considering that ecological restoration and reconstruction are currently advocated worldwide, it is critical to study the control effects of parasitoid wasps on striped rice stem borers to reduce the application of pesticides for sustainable agriculture.

Nitrous oxide (N_2_O) and methane (CH_4_) are two major greenhouse gases (GHGs), and paddy fields are one of the major sources. Methane emissions from paddy fields account for approximately 12% of the global anthropogenic CH_4_ emissions (Intergovernmental Panel on Climate Change [IPCC], 2013). Rice plants are a vital factor affecting the production and emissions of N_2_O and CH_4_ (Yu et al., 1997). Nitrous oxide emissions from soils are regulated by rice plants mainly through changing rhizosphere pH, redox potential, and soil structure (Xu et al., 2000; Yan et al., 2000). Over 80% of the total N_2_O emissions are transported via rice plants in submerged paddy fields, while less than 20% are transported via aerial plant organs (Seiler et al., 1984; Conrad, 1996; Yan et al., 2000). Moreover, the CH_4_ transported through the plants during the rice growing season accounts for nearly 90% of the total CH_4_ emissions from paddy fields (Cicerone et al., 1983; Banker et al., 1995).

Given the critical role that the root aerenchyma tissues play in the GHG transportation from rhizosphere to atmosphere (Lu et al., 1999), and the major impact of root exudation on microbial processes such as nitrification, denitrification, methanogenesis and methanotrophy (Jia and Cai, 2003), the damage inflicted upon rice plants by striped rice stem borers will undoubtedly affect the N_2_O and CH_4_ emissions from paddy fields. Although the effects of striped rice stem borers and parasitoid wasps on the growth of rice plants have been well documented, and parasitoid wasps have been proved to be highly effective in controlling the outbreak of stem borers while reducing the environmental pollution caused by chemical pesticides, their impacts on GHG emissions have been rarely studied. Therefore, this study evaluated the control effects of *Chelonus munakatae* (Munakata) against *C. suppressalis*, as well as their impacts on N_2_O and CH_4_ emissions from a double-rice cropping system, aiming to provide a theoretical basis for promoting this biological pest control method in the future. The hypothesis was that *C. munakatae* could effectively control *C. suppressalis*, and their interaction may have significant effects on N_2_O and CH_4_ emissions from paddy soils.

## Materials and Methods

### Experimental Site

The experimental site is located in Huazhong Agricultural University, Wuhan City, Hubei Province, China (30°28′21″N latitude, 114°20′48″E longitude; 28 m above sea level). It has a subtropical monsoon climate with an average annual temperature of 23.6°C and an average annual precipitation of 906.8 mm. Field and pot experiments were simultaneously carried out with a double-rice cropping system (*Oryza sativa* L.; early rice LY287 and late rice J036) in 2018. The main soil properties before the experiments were examined by measuring a composite sample derived from mixing and homogenizing equal amounts of soil samples collected from each plot or each pot in May 2018 (Supplementary Table S1).

### Experimental Design and Field Management

Three treatments including no insect (NI), striped rice stem borers (*C. suppressalis*) (CS), and striped rice stem borers (*C. suppressalis*) + parasitoid wasps (*C. munakatae*) (CS+CM) were applied following a random complete block design with three replications for both field and pot experiments. The eggs of *C. suppressalis* were reared in advance in the lab until they developed into second-instar larvae, and then placed evenly on the rice plants under CS and CS+CM treatments (80 larvae per plot in field experiment and 12 larvae per pot in pot experiment) successively on May 16th 2018 for early rice and August 20th in 2018 for late rice. For CS+CM treatment, the eggs of *C. suppressalis* were exposed to the mature wasps of *C. munakatae* at the beginning and only the ones with wasp eggs laid inside were kept for later rearing.

The experimental field was 216.09 m^2^ (14.7 × 14.7 m) in area, and the area of each plot was 8.41 m^2^ (2.9 × 2.9 m). The plots were separated from each other by 50 cm high and 50 cm wide ridges. Water leakage and fertilizer transference were prevented by covering the ridges with plastic films and digging 1 m wide ditches around each plot. Besides, supporting frames (3 × 3 × 3 m) over each plot were covered by 100 mesh nets to prevent the transference of insects across different plots. As for pot experiment, the soil was obtained by mixing and homogenizing equal amounts of silty and sandy soils followed by sieving (2 mm). Each pot (0.8 × 0.6 × 0.5 m) contained 80 kg soil, which was then watered and compacted. Similar to the field experiment, each pot was covered by a 100 mesh net to prevent insect spreading.

Rice seedlings were manually transplanted on April 23rd (early rice) and July 25th (late rice). For field experiment, the field was plowed by a rotary tiller and submerged for a week to exterminate the native pests, followed by seedling transplanting at the rate of 7.0 × 10^5^ seedlings ha^–1^ (144 hills per plot with 3 plants hill^–1^). For pot experiment, rice seedlings were transplanted at the rate of 18 seedlings per pot (6 hills per pot with 3 plants hill^–1^). The weeding, irrigation and fertilization regimes were the same for both field and pot experiments. No chemical pesticides or herbicides were applied during the rice growing seasons, and weeds were removed manually. Except for being drained for 2 weeks, respectively, before tillering and harvest stages, the paddy field was irrigated regularly to keep the soil surface wet. As for fertilization, compound fertilizer (N:P_2_O_5_:K_2_O = 15%:15%:15%), urea (N of 46%) and potassium chloride (K_2_O of 60%) were applied to provide 195 kg N ha^–1^, 97.5 kg P_2_O_5_ ha^–1^ and 195 kg K_2_O ha^–1^ for early rice and 240 kg N ha^–1^, 120 kg P_2_O_5_ ha^–1^ and 240 kg K_2_O ha^–1^ for late rice throughout the rice growing season. During the process, P fertilizer was only applied as basal treatment. Half of the K fertilizer was applied as the basal fertilizer, while the other half was applied as a top dressing at the jointing stage. As for N fertilizer, 50, 30, and 20% were applied 2 days before the seedling transplanting, at the tillering stage and at the jointing stage, respectively.

#### Analysis of Plant Growth and Rice Yield

After the insect placement, the ratios of dead tillers of rice plants under CS and CS+CM treatments were measured by calculating the average ratio of dead tillers to total tillers of 36 hills of rice plants around the insect placement sites in each plot. The measurements were carried out every 2 days until they reached stable values. The calculating equation was as below,

(1) D⁢R=D⁢T/T⁢T×100%

where *DR* is the ratio of dead tillers, *DT* is the number of dead tillers, and *TT* is the total number of tillers per plant.

The vitality of rice roots at the harvest stage (July 8th for early rice and October 28th for late rice) was determined by bleeding sap flow method (Carvajal et al., 1996). One hill of rice plants was selected in each plot, and it was made sure that the growth status of the selected plants under the same treatment was similar to each other. With the tip cut off, the rest part of the roots was wrapped in water-proof sealing bags and the bleeding sap flow from the injured roots was then collected and weighed. In addition, another rice plant was selected in each plot at the harvest stage, and it was made sure that the growth status of the selected plants under the same treatment was similar to each other. This whole plant was dug out and washed clean, and then scanned by a root scanner (Fansheng, WinRHIZO, China) to analyze the root morphology. Moreover, the rice grain yield was evaluated at the harvest stage for both early and late rice in field experiment.

### Gas Sampling and Analysis

The N_2_O and CH_4_ fluxes from paddy soil were determined by static chamber-gas chromatography method (Li et al., 2013). In pot experiment, box chambers (0.8 × 0.6 × 0.5 m or 0.8 × 0.6 × 1 m depending on plant height) made of stainless steel and covered with heat-insulation layers were used. A thermometer for temperature monitoring, four fans for air mixing and a vent tube for pressure equilibration were installed inside the chamber. Sampling was conducted every 7 days and every time after N fertilization throughout the rice growing seasons, with four samples successively collected per pot at the interval of 10 min (0, 10, 20, and 30 min) from the chamber headspace using a 30-mL gas-tight syringe. Gas samples were immediately transferred to 30-mL air-evacuated gas-tight glass vials for further analysis.

Nitrous oxide and CH_4_ concentrations were measured by a gas chromatography (Shimadzu, GC-14B, Japan), which was equipped with a stainless-steel column (Porapack N, length × inner diameter of 3 m × 2 mm), an electron capture detector (ECD) and a flame ionization detector (FID). The flow rates of the carrier (N_2_), fuel (H_2_) and supporting gas (zero air) were 30 mL min^–1^, 25 mL min^–1^ and 300 mL min^–1^, respectively, and the column and injector temperatures were adjusted to 50 and 100°C, respectively. The temperature of ECD for N_2_O analysis was adjusted to 330°C and that of FID for CH_4_ analysis was set to 200°C. The N_2_O and CH_4_ fluxes were calculated following the equation below (Zheng et al., 2006),

(2) F=ρ×h×(d⁢c/d⁢t)×273/(273+T)

where *F* is the flux of N_2_O or CH_4_ (mg m^–2^ h^–1^), ρ is the density of N_2_O or CH_4_ standard gas (mg m^–3^), *h* is the height of the chamber headspace (m), *dc/dt* is the increasing rate of gas concentration in the chamber (mg m^–3^ h^–1^), and *T* is the mean temperature of the chamber in the unit of°C. The seasonal cumulative N_2_O and CH_4_ emissions per pot were computed according to Li et al. (2013) as shown below,

(3) $$C\,E\,P_{N_{2}\,O}=\sum_{i=1}^{n}\left\{\left(F_{i}+F_{i+1}\right)\times D_{i}\right\}/2\times 24\times 0.48$$

(4) $$C\,E\,P_{C\,H_{4}}=\sum_{i=1}^{n-1}\left\{\left(F_{i}+F_{i+1}\right)\times D_{i}\right\}/2\times 24\times 0.48/1000$$

where *CEP_*N*__2__*O*_* is the seasonal cumulative N_2_O emissions per pot (mg pot^–1^ season^–1^), *CEP_*CH*__4_* is the seasonal cumulative CH_4_ emissions per pot (g pot^–1^ season^–1^), *F*_*i*_ and *F_*i+*__1_* are the gas fluxes measured on two adjacent sampling dates (mg m^–2^ h^–1^), *D*_*i*_ is the length of the *i*th sampling interval (d), *n* is the total number of sampling intervals and 0.48 is the surface area of the pot (m^2^).

The global warming potential (GWP) of N_2_O and CH_4_ is 298 and 34 times higher than that of carbon dioxide (CO_2_) over a 100-year period (Intergovernmental Panel on Climate Change [IPCC], 2013). Hence, the total seasonal GWP of N_2_O and CH_4_ was calculated as below,

(5) $$G\,W\,P=\left(C\,E_{N_{2}\,O}\times 298+C\,E_{C\,H_{4}}\times 34\right)/1000$$

(6) $$C\,E_{N_{2}\,O}=C\,E\,P_{N_{2}\,O}/0.48/100$$

(7) $$C\,E_{C\,H_{4}}=C\,E\,P_{C\,H_{4}}/0.48\times 10$$

where *GWP* is the total seasonal GWP of N_2_O and CH_4_ (t CO_2_-eq hm^–2^ season^–1^), *CE_*N*__2__*O*_* is the seasonal cumulative N_2_O emissions (kg hm^–2^ season^–1^), *CE_*CH*__4_* is the seasonal cumulative CH_4_ emissions (kg hm^–2^ season^–1^), and *CEP_*N*__2__*O*_* and *CEP_*CH*__4_* are the same as mentioned above.

### Soil Sampling and Chemical Analysis

Two soil samples were randomly taken from each pot during the five rice growing stages of seedling, tillering, booting, full heading and harvest (April 24th, May 12th, May 26th, June 3rd and July 8th for early rice, respectively; July 26th, August 19th, September 8th, September 25th and October 28th for late rice, respectively) from the depth of 0–5 and 0–20 cm, respectively. The two samples from the same pot and of the same depth were then immediately mixed and homogenized after the removal of stones and roots. The samples of 0–5 cm deep soil were stored at –20°C for chemical analysis, while the samples of 0–20 cm deep soil were stored at –80°C for further biological analysis.

The concentrations of soil ammonium-nitrogen (NH_4_^+^-N) and nitrate-nitrogen (NO_3_^–^-N) were measured by ultraviolet spectrophotometric method and colorimetric method with alpha-Naphthol Blue, respectively (Keeney and Nelson, 1982; Baethgen and Alley, 1989). Soil dissolved organic carbon (DOC) was extracted from soil-water solution using suction filtration, and then measured according to the Walkley–Black method (Allison, 1965).

### Absolute Real-Time Quantitative PCR Analysis

Total genomic DNA was extracted from 0–20 cm deep soil samples collected at the tillering, booting, full heading and harvest stages (as mentioned above) using the Fast DNA SPIN Kit For Soil (MP Biomedicals LLC, United States) and purified by the QIAquick Gel Extraction Kit (QIAGEN Ltd., Shanghai, China). The genomic DNA was then analyzed using a NanoDrop spectrophotometer (NanoDrop Technologies Inc., Wilmington, DE, United States), and the A260/A280 ratios were determined to be above 1.8 and the A260/A230 ratios above 2.0.

Absolute real-time quantitative PCR (RT-qPCR) was performed using IQ5 Real-Time PCR detection system (Bio-Rad Laboratories Inc., United States) in 96-well PCR optical plates in triplicate per sample with *AOA-amoA*, *AOB-amoA*, *nirS*, *nirK*, *pmoA*, and *mcrA* as the target genes, following the PCR protocols shown in Supplementary Table S2. Standards for all assays were prepared from clones containing known numbers of DNA copies of the target genes as a plasmid insert (Angel et al., 2011). Standards were then diluted and used for the construction of standard curves in each reaction. Each reaction was composed of 12.5 μL of 2 × SYBR Premix Ex Taq TM (TaKaRa Inc., China), 0.5 μL of 10 pmol μL^–1^ appropriate forward and reverse primers, respectively, and 2 μL of 10-fold diluted template DNA in a final volume of 25 μL. Melting curve analysis was performed to examine the specificity of the amplified products. To avoid PCR inhibition, proper dilution factor of the template DNA was determined in advance by running qPCR with different dilutions of template DNA.

### Statistical Analysis

Statistical analyses were carried out using SPSS (SPSS Inc., Chicago, IL, United States; Version 19.0) and Statistix (Analytical Software Inc., Tallahassee, FL, United States; Version 9.0). Normality and variance homogeneity of data were tested beforehand, and the qPCR datasets were log transformed to meet the requirements for analysis of variance (ANOVA). One-way ANOVA and two-way repeated measures ANOVA were conducted to analyze the data obtained only at harvest stages and at different growing stages, respectively, followed by least significant difference (LSD) test when an ANOVA result was significant. All levels of significance were defined at *P* ≤ 0.05. All plots were drawn with Origin (Origin Lab Corporation, United States; Version 2016).

To elucidate the dynamic mechanisms of GHGs and their relationships with rice plants, soil chemical properties and GHG-related microorganisms, principal component analysis (PCA) was carried out using Canoco (Biometris-Plant Research International, the Netherlands; Version 5.0). GHGs (denoted by red hollow arrow) were represented by the total seasonal GWP of N_2_O and CH_4_. The growth of rice plants (denoted by green arrows) was represented by the attributes of root morphology and vitality as well as the ratio of dead tillers. The soil chemical properties (denoted by red solid arrows) were represented by the NH_4_^+^-N and NO_3_^–^-N concentrations. The GHG-related soil microorganisms (denoted by black arrows) were represented by the abundance of functional genes including *AOA-amoA*, *AOB-amoA*, *nirS*, *nirK*, *pmoA*, and *mcrA*.

## Results

### Plant Growth and Rice Grain Yield

Striped stem borers significantly inhibited the growth and vigor of rice plants. Compared with CS, CS+CM significantly decreased the ratio of dead tillers by 32.5–33.6% (Supplementary Table S3). Moreover, compared with NI, CS significantly reduced the root length, surface area, volume and bleeding sap flow of both early and late rice by 38.0–46.1, 37.4–48.8, 31.9–53.4, and 32.8–47.5%, respectively, while CS+CM only significantly decreased the root length, surface area and volume of late rice by 18.1, 43.2, and 18.4%, respectively (Table 1). Compared with CS, CS+CM enhanced the root length, surface area and volume of late rice by 52.1, 10.8, and 74.9%, respectively (Table 1).

**TABLE 1:** Morphology and vitality of rice roots at harvest stage under different treatments in field experiment.

**Stage**	**Treatment**	**Length (cm hill**^–^**^1^)**	**Surface area (cm^2^ hill^–1^)**	**Volume (cm^3^ hill^–1^)**	**Bleeding sap flow (g hill^–1^)**
Early rice	NI	7376.6 ± 712.5 a	1764.2 ± 166.3 a	31.3 ± 4.5 a	2.56 ± 0.20 a
	CS	4575.5 ± 883.9 b	1103.9 ± 221.7 b	21.3 ± 4.6 b	1.72 ± 0.19 b
	CS+CM	6428.8 ± 1519.7 ab	1494.1 ± 301.3 ab	27.7 ± 4.8 ab	1.91 ± 0.19 ab
Late rice	NI	9233.6 ± 629.3 a	2047.1 ± 212.1 a	35.8 ± 4.9 a	1.39 ± 0.36 a
	CS	4973.5 ± 424.5 c	1048.2 ± 27.0 c	16.7 ± 1.3 c	0.73 ± 0.31 b
	CS+CM	7562.4 ± 654.7 b	1161.9 ± 239.5 b	29.2 ± 6.1 b	1.09 ± 0.23 ab

*Different letters in the same column indicate significant differences at the level of 0.05. *NI*, no insect; *CS*, striped rice stem borers; *CS*+*CM*, striped rice stem borers + parasitoid wasps.*

Compared with NI, the insect treatments significantly decreased the grain yield of both early and late rice by 10.3–22.1% (Supplementary Table S4). Particularly, the grain yield of late rice under CS+CM was significantly higher than that under CS by 15.1%, while no significant difference was observed in the grain yield of early rice between CS and CS+CM (Supplementary Table S4).

### N_2_O and CH_4_ Emissions

**FIGURE 1:**
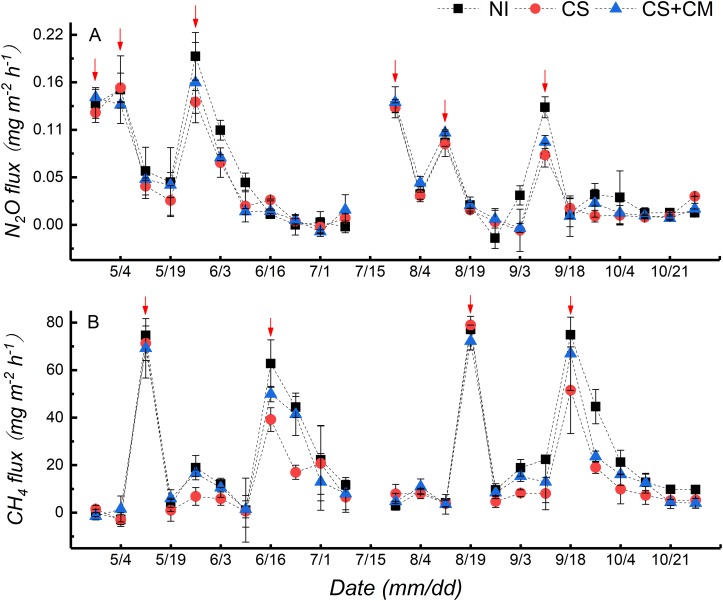
N_2_O **(A)** and CH_4_ **(B)** fluxes from paddy soil under different treatments throughout rice growing season in pot experiment. *NI*, no insect; *CS*, striped rice stem borers; *CS*+*CM*, striped rice stem borers + parasitoid wasps. Red arrows denote the peaks of N_2_O and CH_4_ fluxes.

The variations of N_2_O fluxes throughout the growing seasons were similar between early and late rice (Figure 1A). Three peaks of N_2_O fluxes were observed after the application of N fertilizers and field drying, respectively, at the seedling (April 24th for early rice and July 26th for late rice), tillering (May 4th for early rice and August 12th for late rice) and jointing (May 26th for early rice and September 8th for late rice) stages. The N_2_O fluxes ranged from –0.02 to 0.19 mg m^–2^ h^–1^ under NI, from –0.01 to 0.16 mg m^–2^ h^–1^ under CS, and from –0.01 to 0.16 mg m^–2^ h^–1^ under CS+CM, with the average values of 0.05, 0.04 and 0.05 mg m^–2^ h^–1^, respectively. Furthermore, the seasonal cumulative N_2_O emissions under CS of both early and late rice were significantly lower than those under NI by 17.7–24.6%, while no significant differences were found between NI and CS+CM or between CS and CS+CM (Table 2).

**TABLE 2:** Seasonal cumulative emissions of N_2_O and CH_4_ per pot and total seasonal GWP under different treatments in pot experiment.

**GHG emission**	**Treatment**	**Early rice**	**Late rice**
N_2_O (mg pot^–1^	NI	64.64 ± 2.70 a	43.85 ± 2.37 a
season^–1^)	CS	53.18 ± 4.48 b	33.05 ± 5.46 b
	CS+CM	57.24 ± 6.96 ab	38.17 ± 5.87 ab
CH_4_ (g pot^–1^	NI	22.80 ± 0.94 a	27.91 ± 1.39 a
season^–1^)	CS	14.80 ± 0.94 c	18.81 ± 0.87 c
	CS+CM	19.69 ± 1.61 b	22.56 ± 1.02 b
GWP (t CO_2_-eq hm^–2^	NI	16.55 ± 0.67 a	20.05 ± 0.99 a
season^–1^)	CS	10.81 ± 0.67 c	13.53 ± 0.63 c
	CS+CM	14.30 ± 1.17 b	16.22 ± 0.73 b

*Different letters in the same column indicate significant differences at the level of 0.05. *NI*, no insect; *CS*, striped rice stem borers; *CS*+*CM*, striped rice stem borers + parasitoid wasps.*

The variations of CH_4_ fluxes throughout the growing seasons were similar between early and late rice (Figure 1B). Two peaks were observed at the tillering (May 12th for early and August 19th for late rice) and full heading (June 16th for early rice and September 18th for late rice) stages. The CH_4_ fluxes ranged from –2.92 to 77.14 mg m^–2^ h^–1^ under NI, from –2.95 to 78.98 mg m^–2^ h^–1^ under CS, and from –1.50 to 72.19 mg m^–2^ h^–1^ under CS+CM, with the average values of 23.55, 16.06, and 19.63 mg m^–2^ h^–1^, respectively. Moreover, the seasonal cumulative CH_4_ emissions under insect treatments of both early and late rice were significantly lower than those under NI by 13.6–35.1% (Table 2). However, CS+CM significantly increased the seasonal cumulative CH_4_ emissions by 19.9–33.0% compared with CS (Table 2).

Compared with NI, the insect treatments significantly reduced the total seasonal GWP of N_2_O and CH_4_ from paddy soil by 13.6–34.7% (Table 2). However, the total seasonal GWP of N_2_O and CH_4_ under CS+CM was significantly higher than that under CS by 19.9–32.3% (Table 2).

### Ammonium-N, NO_3_^–^-N and DOC Concentrations

For early rice, both soil NH_4_^+^-N and NO_3_^–^-N concentrations increased and peaked at the booting stage, followed by a dramatic decrease thereafter, while they continuously declined throughout the growing season of late rice (Supplementary Figure S1). Compared with NI, CS significantly increased the soil NH_4_^+^-N concentrations at the later stages of rice growing season by 38.4–88.3%, while nearly no significant difference was observed between NI and CS+CM (Supplementary Figure S1A). Moreover, the soil NH_4_^+^-N concentrations under CS+CM were significantly lower than those under CS by 34.0–36.2% at the later stages of the rice growing season (Supplementary Figure S1A). In contrast, CS significantly decreased the soil NO_3_^–^-N concentrations at the later growing stages of the early rice and at the booting stage of the late rice by 23.7–32.6% relative to NI, and there were no significant differences between NI and CS+CM or between CS and CS+CM (Supplementary Figure S1B).

The DOC concentrations in paddy soil of early and late rice both peaked at the booting stage (Supplementary Figure S2). Furthermore, under CS, the soil DOC concentrations at the later stages of the rice growing season were significantly lower than those under NI by 13.9–39.5%; while under CS+CM, only the DOC concentrations at the booting stage of early rice and full heading stage of late rice were significantly lower than those under NI by 11.0–19.7% (Supplementary Figure S2). No significant difference in DOC concentrations was found between CS and CS+CM (Supplementary Figure S2).

### Abundance of Microbial Functional Genes

**FIGURE 2:**
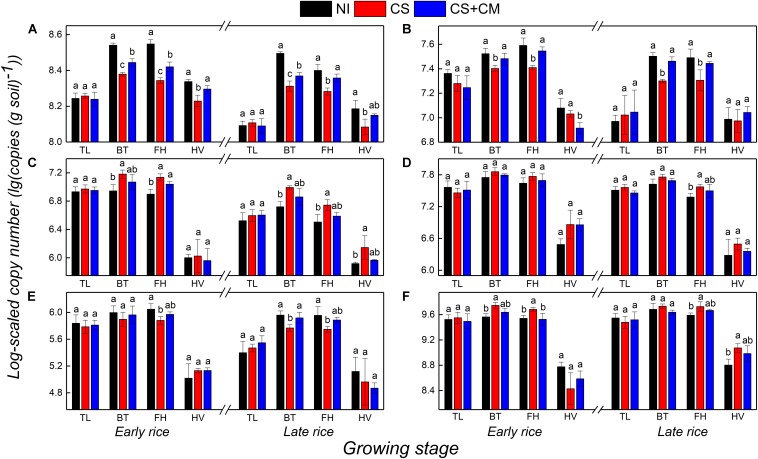
Abundance of *AOA-amoA* **(A)**, *AOB-amoA* **(B)**, *nirS* **(C)**, *nirK* **(D)**, *pmoA* **(E)** and *mcrA* **(F)** genes at log-scale in paddy soil under different treatments at tillering, booting, full heading and harvest stages in pot experiment. *NI*, no insect; *CS*, striped rice stem borers; *CS*+*CM*, striped rice stem borers + parasitoid wasps. TL, tillering stage; BT, booting stage; FH, full heading stage; HV, harvest stage. The abundance of genes was measured in triplicate per sample. Different letters indicate significant differences at the level of 0.05.

The abundance of *AOA-amoA* and *AOB-amoA* genes that regulate the nitrification in paddy soil both peaked at the booting and full heading stages, ranging from 1.92 × 10^8^ to 3.53 × 10^8^ copies (g soil) ^–1^ and 2.00 × 10^7^ to 3.92 × 10^7^ copies (g soil)^–1^, respectively (Figures 2A,B). Moreover, their abundance was significantly affected by the insect treatments (Figures 2A,B). Compared with that under NI, the abundance of *AOA-amoA* at the booting, full heading and harvest stages under CS was 21.0–37.5% lower, while that under CS+CM was reduced by 9.9–25.4%. Besides, CS+CM significantly enhanced the abundance of *AOA-amoA* at the booting, full heading and harvest stages by 13.8–19.4% compared with CS. On the other hand, the abundance of *AOB-amoA* under CS at the booting and full heading stages was significantly lower than that under NI by 24.5–37.1%, whereas nearly no significant difference in the abundance of *AOB-amoA* was observed between NI and CS+CM. Furthermore, compared with CS, CS+CM significantly enhanced the abundance of *AOB-amoA* at the booting and full heading stages by 20.8–45.1%.

The abundance of both *nirS* and *nirK* genes that regulate the denitrification in paddy soil peaked at the booting stages, and ranged from 5.31 × 10^6^ to 1.53 × 10^7^ copies (g soil) ^–1^ and from 4.27 × 10^7^ to 7.31 × 10^7^ copies (g soil) ^–1^, respectively (Figures 2C,D). In addition, the insect treatments only imposed significant impacts on the abundance of *nirS* (Figures 2C,D). The abundance of *nirS* under CS at the booting, full heading and harvest stages was significantly higher than that under NI by 71.3–85.8%, while there were no significant differences between NI and CS+CM or between CS and CS+CM.

The abundance of both *pmoA* and *mcrA* genes, which, respectively, regulate the oxidization and production of CH_4_ in paddy soils, peaked at the booting and full heading stages, ranging from 5.63 × 10^5^ to 1.13 × 10^6^ copies (g soil) ^–1^ and 3.41 × 10^9^ to 5.59 × 10^9^ copies (g soil) ^–1^, respectively (Figures 2E,F). Compared with NI, CS significantly lowered the abundance of *pmoA* at the booting and full heading stages by 32.1–39.7%, while nearly no significant differences were observed between NI and CS+CM or CS and CS+CM (Figure 2E). On the contrary, the abundance of *mcrA* under CS at the booting, full heading and harvest stages was significantly higher than that under NI by 38.3–85.0%, whereas there was nearly no significant difference between NI and CS+CM or CS and CS+CM (Figure 2F).

**FIGURE 3:**
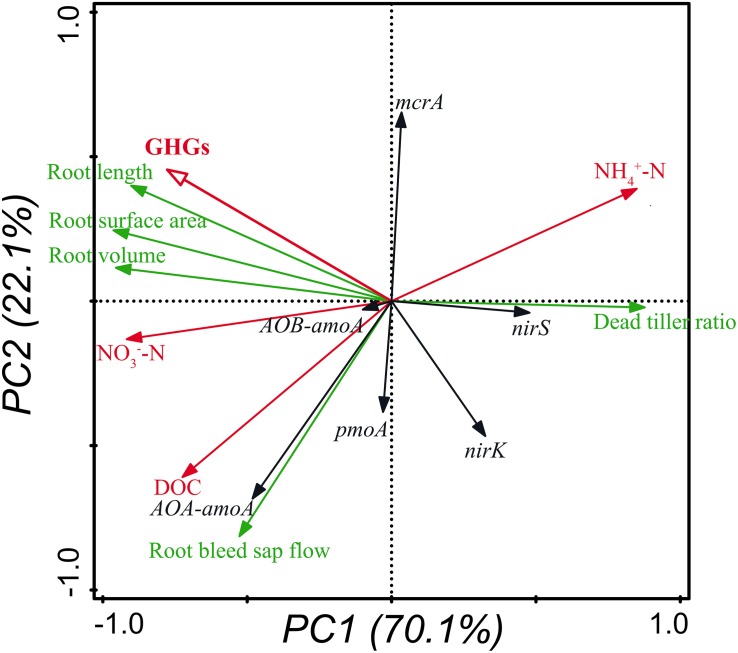
Relationships of GHGs with the growth of rice plants, soil chemical properties and GHG-related microorganisms shown by PCA. GHGs and soil chemical properties are denoted by hollow and solid red arrows, respectively. Attributes of rice plants are denoted by green arrows, and soil microorganisms are denoted by black arrows. The abundance of genes was measured in triplicate per sample.

As shown in the PCA plot, two axes accounted for 70.1 and 22.1% of the variance (Figure 3). GHGs were positively correlated with the morphological indexes of rice roots, soil DOC and NO_3_^–^-N concentrations, and the abundance of nitrifiers harboring *AOB-amoA* and methanogens harboring *mcrA*, while were negatively correlated with the ratio of dead tillers, soil NH_4_^+^-N concentrations, and the abundance of denitrifiers harboring *nirS* and *nirK* and methanotrophs harboring *pmoA* (Figure 3).

## Discussion

### Impacts of *C. suppressalis* and *C. munakatae* on Rice Growth and Grain Yield

Khush and Toenniessen (1991) claimed that stem borer damage could result in a 10–30% loss of total rice grain yield. Our results also showed that *C. suppressalis* significantly decreased rice grain yield (Supplementary Table S4) compared with no insect treatment, possibly owing to the increased ratio of dead tillers (Supplementary Table S3), dead shoots and the resultant formation of dead hearts and white heads caused by *C. suppressalis*. This explanation is supported by previous studies (Pathak, 1968; Dale, 1994), which reported that larval feeding of *C. suppressalis* on rice plants could lead to the yellowing and dying of growing shoots, fewer tillers and more panicles with unfilled grains. In addition, the inhibited growth and vitality of rice roots (Table 1) induced by nutrient consumption and tissue damage in rice plants under the *C. suppressalis* treatment, may limit the uptake and transport of nutrients through the roots and negatively affect the grain filling process, thus reducing grain yield (Pathak et al., 1971).

Moreover, numerous studies have concluded that natural enemies could effectively control the outbreak of pests (Beddington et al., 1978; Settle et al., 1996; Losey and Vaughan, 2006). Our results also indicated that *C. munakatae* is highly effective in controlling *C. suppressalis* to significantly decrease the ratio of dead tillers (Supplementary Table S3), improve the growth of roots (Table 1), and increase the grain yield (Supplementary Table S4). It has been reported that *C. munakatae* can impair the activity of *C. suppressalis* through parasitism, thereby alleviating their damage on rice plants and ultimately enhancing the rice yield (Maki, 1930).

### Impacts of *C. suppressalis* and *C. munakatae* on GHG Emissions and Related Microorganisms

In this study, N_2_O fluxes from paddy soils peaked three times just after the application of N fertilizers and field drying throughout the rice growing season (Figure 1A), suggesting that N_2_O emissions are highly correlated with N fertilization and field drying. Similarly, Yao et al. (2000) and Wang et al. (2011) revealed that N fertilization could stimulate N_2_O emissions by providing more N substrates for the microbial nitrification and denitrification in soils. Moreover, Zhang et al. (2015) concluded that field drying increases N_2_O emissions from paddy fields by facilitating microbial nitrification due to the improvement of soil aeration. As for CH_4_ fluxes from paddy soil in this study, two peaks were observed at the tillering and full heading stages, respectively (Figure 1B). This may be attributed to the increased aerenchyma cells in rice plants at the tillering stage, which could facilitate CH_4_ transport (Bhattacharya et al., 2013) and the production of more root exudates at the full heading stage, and higher CH_4_ production by supplying more nutrients for methanogens (Lu et al., 2000).

This study shows that insect treatments could significantly decrease the fluxes (Figure 1) and seasonal accumulative emissions (Table 2) of N_2_O and CH_4_, as well as the total seasonal GWP (Table 2) compared with no insect treatment, probably because the growth of rice roots and shoots was inhibited by *C. suppressalis* parasitism (Supplementary Table S3 and Table 1), which limited the gas transport through root and shoot aerenchyma. The PCA results also imply that GHGs were highly positively correlated with the growth of rice roots (Figure 3). This conclusion is supported by numerous studies, which suggests that plant-mediated transport is a major pathway for gas efflux in submerged soil (Hosono and Nouchi, 1997; Groot et al., 2005; Reid et al., 2015) and more than 80% of both N_2_O and CH_4_ is emitted through rice plants (Yu et al., 1997; Watanabe et al., 1999; Yan et al., 2000). Similarly, the relatively higher fluxes (Figure 1) and seasonal accumulative emissions (Table 2) of N_2_O and CH_4_ as well as the significantly higher total seasonal GWP (Table 2) under CS+CM than under CS may also be owing to the lower damage on rice plants under CS+CM (Supplementary Table S3 and Table 1) as mentioned above.

As shown by the results of this study, the abundance of both nitrifiers and methanotrophs was significantly lower under the *C. suppressalis* treatment than under no insect treatment (Figures 2A,B,E), whereas that of both denitrifiers and methanogens was significantly higher (Figures 2C,F), which could be explained by the reduction of oxygen released from rice roots due to the root damage under the *C. suppressalis* treatment (Table 1), and the subsequent activation of anaerobic microorganisms including denitrifiers and methanogens. The PCA results also support this speculation, illustrating that the abundance of both nitrifiers harboring *AOA-amoA* and *AOB-amoA* and methanotrophs harboring *pmoA* is positively correlated with the growth and vitality of rice roots (Figure 3).

### Dynamic Mechanisms of GHGs Under Insect Treatment

As shown by the PCA results, GHGs had much higher correlations with the morphological indexes of rice roots than with the GHG-related soil microorganisms (Figure 3), implying that rice roots play a more important role than GHG-related microorganisms in regulating the GHG emissions under insect treatment from paddy soil. This conclusion is also supported by another result: when rice roots were damaged by *C. suppressalis* parasitism (Table 1), the GHG emissions and total seasonal GWP were both significantly reduced (Figure 1 and Table 2), even though the abundance of denitrifiers and methanogens was significantly higher than that under no insect treatment (Figures 2C,F). Similarly, Zhang et al. (2016) observed that the underground part of rice plants is a major contributor to CH_4_ emissions from paddy fields. Das and Baruah (2008) also reported that the length and volume of rice roots are significantly positively correlated with GHG emissions.

Apart from the roots, soil organic carbon (SOC) is another vital factor affecting the GHGs from paddy soil (Tokida et al., 2011), as SOC affects the microbial activity directly by providing substrates and nutrients, or indirectly by altering the oxygen exchange through root by affecting the root growth (Lu et al., 2002; Cai et al., 2016). Jiang et al. (2018) revealed that the contribution of GHG-related microorganisms to GHG emissions is higher than that of root aerenchyma only when the SOC concentrations are high enough. The SOC concentrations in this study were relatively low due to the absence of straw incorporation. Hence, it was the roots rather than the GHG-related microorganisms that play a determining role in the dynamic mechanisms of GHGs from paddy soils.

Although this study provides interesting insight into the dynamic mechanisms of GHGs in paddy fields under insect treatment, a few shortcomings still exist. For example, only the data of 1 year were collected. There was no comparison between the biological and chemical approaches by including chemical pesticide application as a treatment, and no straw was incorporated into the soil. Therefore, further research should be carried out to address these problems to acquire a more thorough understanding of the effects of this biological approach.

## Conclusion

This study focused on the control effects of *C. munakatae* against *C. suppressalis* and their impacts on rice plants and GHG emissions from paddy fields. Compared with no insect treatment, *C. suppressalis* treatment significantly increased the ratio of dead tillers, inhibited the growth and vitality of rice roots and decreased the grain yield. However, the GHG emissions from paddy soil under the *C. suppressalis* treatment were significantly lower than those under no insect treatment, due to the morphological changes of rice roots. *Chelonus munakatae* could effectively control the *C. suppressalis* outbreak and alleviate the pest damage by reducing the ratio of dead tillers and enhancing the rice grain yield without the application of chemical pesticides. The relatively higher GHG emissions under *C. munakatae* + *C. suppressalis* treatment compared with those under *C. suppressalis* treatment alone, were acceptable since they were still significantly lower than those under no insect treatment. Therefore, the prevention and control of *C. suppressalis* outbreak in paddy fields by *C. munakatae* is an effective, environment-friendly and sustainable approach that should be widely promoted.

## Data Availability Statement

The raw data supporting the conclusions of this article has been made available by the authors, without undue reservation, to any qualified researcher (Supplementary Data Sheet S1).

## Author Contributions

CL conceived and designed the research. HZ, DF, and TL provided the experimental data. DF and HZ performed the statistical analysis. DF wrote the manuscript. CL and CC commented on and revised the manuscript.

## Conflict of Interest

The authors declare that the research was conducted in the absence of any commercial or financial relationships that could be construed as a potential conflict of interest.
