# Supplementary material for: Control Effects of Chelonus munakatae Against Chilo suppressalis and Impact on Greenhouse Gas Emissions From Paddy Fields
Source: Front Plant Sci. 2020 Mar 6;11:228. doi: 10.3389/fpls.2020.00228 (PMC7067967; doi:10.3389/fpls.2020.00228)
Supplement: Supplementary file 1 [file Data_Sheet_1.docx]

**Table S1**

Main soil properties before the experiments in 2018

| Experiment | Total P  (g kg-1) | Available P  (mg kg-1) | Total K (g kg-1) | Available K (mg kg-1) | Total N (g kg^-1^） | NO_3_^-^-N (mg kg^-1^) | NH_4_^+^-N (mg kg^-1^) | | pH | Organic matter (mg kg^-1^) |
| --- | --- | --- | --- | --- | --- | --- | --- | --- | --- | --- |
| Pot | 0.46 | 9.88 | 3.15 | 86.35 | 10.96 | 6.78 | | 7.39 | 6.22 | 59.74 |
| Field | 0.51 | 10.59 | 3.56 | 95.67 | 2.63 | 1.71 | | 1.43 | 6.37 | 29.61 |

**Table S2**

Primers and protocols for RT-qPCR

| Target gene | Primers*^a^* | Product (bp) | PCR protocol |
| --- | --- | --- | --- |
| *AOA-amoA* | Arch-amoAF/Arch-amoAR  (Francis et al., 2005) | 635 | 94^°^C for 3 min,  40 cycles of 94^°^C for 30 s, 53^°^C for 1 min, 72^°^C for 1 min,  72^°^C for 10 min (Chen et al., 2008) |
| *AOB-amoA* | amoA-1F/amoA-2R  (McTavish et al., 1993) | 491 | 94^°^C for 3 min,  40 cycles of 94^°^C for 30 s, 55^°^C for 30 s, 72^°^C for 45 s,  72^°^C for 10 min (Chen et al., 2008) |
| *nirS* | cd3AF (Michotey et al., 2000)/  R3cd (Throbäck et al., 2004) | 425 | 94^°^C for 3 min,  40 cycles of 95^°^C for 45 s, 57^°^C for 45 s, 72^°^C for 45 s,  72^°^C for 10 min (Bannert et al., 2011) |
| *nirK* | nirK1F (Henry et al., 2004)/  nirK5R (Braker et al., 1998) | 472 | 94^°^C for 3 min,  40 cycles of 95^°^C for 15 s, 58^°^C for 30 s, 72^°^C for 30 s,  72^°^C for 10 min (Bannert et al., 2011) |
| *pmoA* | mlas-mod-F/mcrA-rev-R  (Roey et al., 2012) | 491 | 94 ℃ for 4 min,  5 cycles of 94^°^C for 30 s, 60^°^C for 45 s, 72^°^C for 30 s,  30 cycles of 94^°^C for 30 s, 55^°^C for 30 s, 72^°^C for 30 s,  72^°^C for 10 min (Roey et al., 2012) |
| *mcrA* | A189F/mb661R  (Mao et al., 2015) | 469 | 95^°^C for 3 min,  10 cycles of 95^°^C for 15 s, 62–53^°^C for 30 s (-1^°^C /cycle), 72^°^C for 30 s,  35 cycles of 95^°^C for 15 s, 52^°^C for 30 s, 72^°^C for 30 s, 85^°^C for 30s,  72^°^C for 10 min (Mao et al., 2015) |

*^a^* F stands for forward primer and R stands for reverse primer.

**Table S3**

Ratio of dead tillers of the rice plants (%) under CS and CS+CM treatments in field experiment

| Treatment | Early rice | Late rice |
| --- | --- | --- |
| CS | 21.31±1.58 a | 20.24±4.77 a |
| CS+CM | 14.39±2.08 b | 13.44±2.94 b |

Different letters in the same column indicate significant differences at the level of 0.05. *CS*, striped rice stem borers; *CS+CM*, striped rice stem borers + parasitoid wasps.

**Table S4**

Rice grain yield (t hm^-2^) under different treatments in field experiment

| Treatment | Early rice | Late rice |
| --- | --- | --- |
| NI | 7.08±0.30 a | 6.02±0.10 a |
| CS | 5.55±0.28 b | 4.69±0.19 c |
| CS+CM | 5.85±0.28 b | 5.40±0.39 b |

Different letters in the same column indicate significant differences at the level of 0.05. *NI*, no insect; *CS*, striped rice stem borers; *CS+CM*, striped rice stem borers + parasitoid wasps.

**

**

**Fig. S1** Soil NH_4_^+^-N (A) and NO_3_^-^-N (B) concentrations under different treatments throughout rice growing season in pot experiment. *NI*, no insect; *CS*, striped rice stem borers; *CS+CM*, striped rice stem borers + parasitoid wasps. SD, seedling stage; TL, tillering stage; BT, booting stage; FH, full heading stage; HV, harvest stage. Different letters indicate significant differences at the level of 0.05.





**Fig. S2** Soil DOC concentrations under different treatments throughout rice growing season in pot experiment in 2018. *NI*, no insect; *CS*, striped rice stem borers; *CS+CM*, striped rice stem borers + parasitoid wasps. SD, seedling stage; TL, tillering stage; BT, booting stage; FH, full heading stage; HV, harvest stage. Different letters indicate significant differences at the level of 0.05.

**References**

Bannert, A., Kleineidam, K., Wissing, L., Mueller-Niggemann, C., Vogelsang, V., and Welzl, G. (2011). Changes in diversity and functional gene abundances of microbial communities involved in nitrogen fixation, nitrification, and denitrification in a tidal wetland versus paddy soils cultivated for different time periods. Appl. Environ. Microbiol. 77, 6109–6116. doi: 10.1128/aem.01751-10

Braker, G., Fesefeldt, A., and Witzel, K. P. (1998). Development of PCR primer systems for amplification of nitrite reductase genes (*nirK* and *nirS*) to detect denitrifying bacteria in environmental samples. Appl. Environ. Microbiol. 64, 3769–3775. doi: 10.1128/aem.64.10.3769-3775.1998

Chen, X. P., Zhu, Y. G., Xia, Y., Shen, J. P., and He, J. Z. (2008). Ammonia−oxidizing archaea: important players in paddy rhizosphere soil. Environ. Microbiol. 10, 1978–1987. doi: 10.1111/j.1462-2920.2008.01613.x

Francis, C. A., Santoro, A. E., Oakley, B. B., Beman, J. M., and Roberts, K. J. (2005). Ubiquity and diversity of ammoniaoxidizing archaea in water columns and sediments of the ocean. Proc. Natl. Acad. Sci. U.S.A. 102, 14683–14688. doi: 10.1073/pnas.0506625102

Henry, S., Ezékiel, B., López-Gutiérrez, J. C., Martin-Laurent, F., Brauman, A., and Philippot, L. (2004). Quantification of denitrifying bacteria in soils by *nirK* gene targeted real-time PCR. J. Microbiol. Methods 59, 327–335. doi: 10.1016/j.mimet.2004.07.002

Mao, T. T., Yin, R., and Deng, H. (2015). Effects of copper on methane emission, methanogens and methanotrophs in the rhizosphere and bulk soil of rice paddy. Catena 133, 233–240. doi: 10.1016/j.catena.2015.05.024

Mctavish, H., Fuchs, J. A., and Hooper, A. B. (1993). Sequence of the gene coding for ammonia monooxygenase in Nitrosomonas europaea. J. Bacteriol. 175, 2436–2444. doi: 10.1128/jb.175.8.2436-2444.1993

Michotey, V., Mejean, V., and Bonin, P. (2000). Comparison of methods for quantification of cytochrome cd1-denitrifying bacteria in environmental marine samples. Appl. Environ. Microbiol. 66, 1564–1571. doi: 10.1128/aem.66.4.1564-1571.2000

Roey, A., Peter, C., and Ralf, C. (2012). Methanogenic archaea are globally ubiquitous in aerated soils and become active under wet anoxic conditions. ISME J. 6, 847–862.

Throbäck, I. N., Enwall, K., Jarvis, A., and Halli, S. (2004). Reassessing PCR primers targeting *nirS*, *nirK* and *nosZ* genes for community surveys of denitrifying bacteria with DGGE. FEMS Microbiol. Ecol. 49, 401–417. doi: 10.1016/j.femsec.2004.04.011
